# 576. Community-based PrEP delivery in urban US settings: the first US mobile retail pharmacy

**DOI:** 10.1093/ofid/ofaf695.185

**Published:** 2026-01-11

**Authors:** Sheela Shenoi, Angela Di Paola, Adati Tarfa, Cynthia Frank, Alysse Schultheis, Ralph P Brooks, Sandra Ann Springer

**Affiliations:** Yale University, New Haven, CT; Yale University, New Haven, CT; Yale University, New Haven, CT; Yale School of Medicine, New Haven, Connecticut; Yale School of Medicine, New Haven, Connecticut; Yale School of Medicine - AIDS Program, New Haven, Connecticut; Yale University, New Haven, CT

## Abstract

**Background:**

PrEP implementation has lagged in the US, requiring innovative models of PrEP delivery. We present pilot data on implementation of community-based PrEP using the first mobile retail pharmacy clinic model in the US.

**Methods:**

A team of community health workers, APRN, phlebotomist, and pharmacist in a mobile pharmacy clinic (MPC) visit urban sites as directed by local health departments, offering whole person health care including primary care, substance use disorder and HIV services. Persons prescribed PrEP fill prescriptions at the attached mobile retail pharmacy.

**Results:**

Among 578 community members who received care through the MPC in urban centers, the median age was 49 (IQR 38-58), 300 (51.9%) identified as men, 273 (47.2%) were Caucasian, 253 (43.8%) were unstably housed or unhoused, 60 (10.4%) had alcohol use disorder, and 113 (19.6%) had substance use disorder. Among 272 (47.1%) who agreed to undergo rapid HIV testing, 1 person tested positive. Among 271 (46.9%) who were PrEP eligible, 10 (3.7%) initiated PrEP. Among PrEP initiators, 9 initiated oral PrEP and 1 initiated injectable PrEP, and 4 (40%) started PrEP on the same day as the visit to the mobile clinic. Among PrEP initiators, 5 (50%) were retained at 4 months.

**Conclusion:**

Community-based PrEP delivery using a mobile pharmacy and clinic is feasible in urban US settings. Incorporation of a mobile retail pharmacy may facilitate PrEP implementation.

**Disclosures:**

Sheela Shenoi, MD MPH, Merck and Company, Inc.: My spouse worked for Merck 1997-2007 and retains stock in his retirement account. There is no conflict of interest, but included for full disclosure.

